# St. Lawrence County Medical Society

**Published:** 1869-01

**Authors:** R. R. Sherman

**Affiliations:** Sec’y.


### St. Lawrence County Medical Society.

This Society held its Annual Meeting at Potsdam Junction, the 17th instant, and elected the following officers for the ensuing year:

President, Dr. S. L. Parmerlee; Vice President, Dr. J. H. Jackson; Treasurer Dr. H. A. Boland; Secretary, Dr. R. R. Sherman.

Censors—Drs. S. L. Parmerlee, E. H. Bridges and C. C. Bates.

Delegates to the State Medical Society—Drs. B. F. Sherman, Z. B. Bridges and Caleb Puice.

Discussionists—Drs. E. H. Bridges, Robert Morris, E. G. Seymour and D. McFalls.

Essayists—Drs. W. H. Cruickshank, E. C. Walsh and B. F. Drury.

Committee of Intelligence—Drs. B. F. Sherman, C. C. Bates and C. Puice.

The Semi-Annual Meeting will be held at Governeur, the second Tuesday in June next.

Subjects for discussion—Anatomy and Physiology of the Kidneys and Bright's Disease.

R. R. Sherman, Sec'y.

